# Regulatory Networks in Pollen Development under Cold Stress

**DOI:** 10.3389/fpls.2016.00402

**Published:** 2016-03-31

**Authors:** Kamal D. Sharma, Harsh Nayyar

**Affiliations:** ^1^Department of Agricultural Biotechnology, Chaudhary Sarwan Kumar Himachal Pradesh Agricultural UniversityPalampur, India; ^2^Department of Botany, Panjab UniversityChandigarh, India

**Keywords:** cold stress, anther, pollen development, pollen sterility, abscisic acid signaling, bioactive gibberellins, sugar metabolism

## Abstract

Cold stress modifies anthers’ metabolic pathways to induce pollen sterility. Cold-tolerant plants, unlike the susceptible ones, produce high proportion of viable pollen. Anthers in susceptible plants, when exposed to cold stress, increase abscisic acid (ABA) metabolism and reduce ABA catabolism. Increased ABA negatively regulates expression of tapetum cell wall bound invertase and monosaccharide transport genes resulting in distorted carbohydrate pool in anther. Cold-stress also reduces endogenous levels of the bioactive gibberellins (GAs), GA_4_ and GA_7_, in susceptible anthers by repression of the GA biosynthesis genes. Here, we discuss recent findings on mechanisms of cold susceptibility in anthers which determine pollen sterility. We also discuss differences in regulatory pathways between cold-stressed anthers of susceptible and tolerant plants that decide pollen sterility or viability.

## Introduction

**FIGURE 1:**
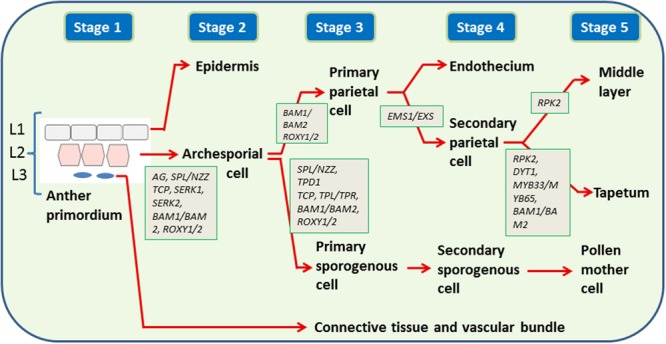
**Genes regulating cell differentiation in early stages (1–5) of anther development.** Three cell layers (L1, L2, L3) present in anther primordium divide to form: L1: epidermis (E), L2: endothecium (En), middle layer (ML), tapetum (T), and pollen mother cell (PMC), L3: connective tissue and vascular bundle (C). The development of L2 to En, ML, T, and PMC proceed through several intermediates, e.g., primary parietal cell, archesporial cell, secondary parietal cell, primary sporogenous cell, secondary sporogenous cell. The genes are shown in italics. *AG*: *AGAMOUS, SEP*: *SEPTELLA, BAM*: *BARELY ANY MERISTEM, ROXY1*/2: *Arabidopsis CC-TYPE GLUTAREDOXINS, SPL*/*NZZ*: *SPOROCYTELESS*/*NOZZLE, TCP*: *TEOSINTE BRANCHED1*/*CYCLOIDEA*/*PCF* transcription factors, *SERK*: *SOMATIC EMBRYOGENESIS RECEPTOR-LIKE KINASE, EMS1/EXS*: *EXCESS MICROSPOROCYTES1*/*EXTRA SPOROGENOUS CELLS, RPK2*: *RECEPTOR-LIKE PROTEIN KINASE2, DYT1*: *DYSFUNCTIONAL TAPETUM1, TPD1*: *TAPETAL DETERMINANT 1, TPL*/*TPR*: *TOPLESS/TOPLESS-RELATED*.

Pollen development within anthers is a well-studied phenomenon (Zhang and Yang, 2014; Gómez et al., 2015). The anthers develop from anther primordia which contain three cell layers, L1, L2, and L3. The primordium layers differentiate into diverse cell types with L1 forming the epidermis, L2 the archesporial and primary parietal cells (PP) and L3 the vascular and connective tissues (**Figure 1**). The archesporial cells divide to form primary sporogenous cells (Sp; Wilson and Zhang, 2009). The Sp form meiocytes through a series of intermediate cell types whereas the PP form tapetum, middle cell layer and endothecium (Wilson and Zhang, 2009; Zhang and Yang, 2014). Molecular switches and signaling pathways in specification of tapetum and microsporocyte cells within the anther have been reviewed recently (Chang et al., 2011; Zhang and Yang, 2014). Within each anther lobe, *SPOROCYTELESS/NOZZLE* (*SPL/NZZ*) gene [a putative novel transcription factor (TF)], which acts downstream of the *AGAMOUS* (*AG*) gene is the regulator of sporogenesis (Yang et al., 1999; Ito et al., 2004; Liu et al., 2009, **Figure 1**). This gene is expressed only in L2 layer (Yang et al., 1999). The important regulators for the formation of PP are the genes *BARELY ANY MERISTEM1* (*BAM1*) and *BAM2 Leucine-rich repeat receptor like Kinases* (Kelliher et al., 2014). In the absence of these genes (the *bam1/2* double mutant anthers), the inner three somatic cell layers are replaced by pollen mother cells (PMC) like cells because of SPL expression in somatic cell layers (Hord et al., 2006). The redox state of the cells is also considered a factor to determine fate of the germ cell and tapetum (Zhang and Yang, 2014) owing to the evidences that the redox genes *ROXY1* (*Arabidopsis CC-TYPE GLUTAREDOXINS1*) and *ROXY2* express in lobe primordia of *Arabidopsis* anthers at a time when the archesporial cell differentiates into sporogenous cells (Rozema et al., 2001), and the double-mutants for these genes were male sterile with early defects in anther lobe formation and non-formation of PMC (Rozema et al., 2001). The *ROXY1* and *ROXY2* are also expressed in PMC and somatic cell layers before meiosis.

The separation of the somatic cells and the meiocytes is important to explore molecular mechanisms governing development of both types of tissues in anthers. This, however, was not possible until the discovery of laser microdissection (LM) assay (Nakazono et al., 2003), a technique that later led to the separation of transcriptomes of pollen and tapetum cells in rice anthers, thereby leading to identification of networks of genes that operate during meiosis (Aya et al., 2011). Several meiosis-specific gene sub-networks, “such as transition from mitosis into meiosis (MEL1, MEL2, OsAML1, RNA polymerase, SET domain, PWWP domain, double-stranded RNA binding, ribonucleoprotein), homologous pairing (PAIR1), synapsis (PAIR2, ZEP1, PAIR3); meiotic replication and chromosome structure control (OsPOLE1, Flap endonuclease, OsRAD21-4, OsSMC2, OsRPA2C); meiotic recombination (DMC1A, DMC1B, BRCA1-associated protein, OsRAD17, RNA helicase, SNF2, OsMER3, OsMSH4, OsMSH5) and meiotic progression (OsSDS, Cyclin A, Cyclin B, Cyclin D, CUL1, SKP1B, OsMMD1, OsCDC20)” were identified (Aya et al., 2011). Like pollen meiosis, pollen mitosis is also a poorly understood process. Pollen mitosis is disrupted in plants with mutated RING-finger E3 ligases, RING-H2 group F 1a (RHF1a) and RHF2a (Liu et al., 2008). These genes play role in *Arabidopsis* pollen mitosis I (PM I) and probably in pollen mitosis II (PM II). In *rhf1a rhf2a* double mutant, 30–40% microspores fail to go through PM I and 20–30% of the surviving microspores fail to undergo PM II (Liu et al., 2008). RHF1a interacts directly with a cyclin-dependent kinase inhibitor ICK4/KRP6 (Interactors of Cdc2 Kinase 4/Kip-related protein 6), which is a negative regulator of mitosis (Liu et al., 2008). In contrast to mitosis where *ICK4/KRP6* transcription is turned off, it is switched on during meiosis (Liu et al., 2008). In addition to this, *Arabidopsis* transcription initiation factor TFIIB-related protein BRP4 is also involved in the regulation of mitotic cell-cycle progression during male gametogenesis (Qin et al., 2014). Lowered expression of this gene results in arrest of mitotic division, as evident from knockdown mutants developed using RNA interference construct (Qin et al., 2014). Furthermore, *ORC6*, a gene encoding a subunit of the origin recognition complex, acts downstream of *BRP4* (Qin et al., 2014). The discussion on programmed death of tapetum, pollen development, exine or intine formation, etc. is out of preview of this article and these have been described elsewhere (Wilson and Zhang, 2009; Parish and Li, 2010; Chang et al., 2011; Quilichini et al., 2014; Zhang and Yang, 2014), however, role of gibberellic acid (GA) needs mention, as this mechanism is disrupted by cold stress (CS) in susceptible genotypes.

## Gibberellic Acid in Pollen Development

Gibberellic acid is a relatively well-studied growth regulator for its role in anther and pollen development. GAs are vital to anther development as well as pollen viability (Kwon et al., 2015), and GA signaling predominantly works in tapetal cells (Plackett et al., 2011). GA regulates tapetum differentiation as well as initiation of tapetum programmed cell death (PCD) via GA-regulated myeloblastosis (GAMYB), a gibberellin-regulated transcriptional activator (Plackett et al., 2014). Enzymes, GA oxidases and GA hydroxylases, catalyze late steps in synthesis of active GAs. Loss of GA20 oxidase activity arrests anther developmental and tapetum does not degrade (Plackett et al., 2011). GA interacts with receptor GID1 (GA INSENSITIVE DWARF1) and the complex binds to the GRAS family protein DELLA (Plackett et al., 2011; Davière and Achard, 2016), a protein that is essential for pollen development in monocots as well as dicots (Plackett et al., 2014; Davière and Achard, 2016). The GRAS gene family is comprised of three members, i.e., GAI (GIBBERELLIN-INSENSITIVE), RGA (REPRESSOR of ga1–3) and SCR (SCARECROW-LIKE) whereas DELLA, a subfamily of GRAS has five members in *Arabidopsis*: GAI, RGA, RGA-LIKE 1 (RGL1), RGL2, and RGL3. The ubiquitination of GID1 and DELLA complex by SCF-E3 ubiquitin ligase leads to proteolysis of DELLA and activation of GA signaling targets (Ueguchi-Tanaka et al., 2007; Plackett et al., 2011). A casein kinase I (CK1) protein called Rice early flowering1 (EL1) phosphorylates DELLA protein SLR1 (slender rice 1) in rice to negatively regulate gibberellin signaling (Dai and Xue, 2010). Recent evidence suggests that the modulation of GA activity by EL1 is essential for anther development and pollen viability as *el1* mutants were early flowering but with low fertility (Kwon et al., 2015). GAMYB appears to be the key TF in GA signaling pathway. It activates the expression of CYP703A3, KAR, and other genes involved in the synthesis of sporopollenin and is essential for anther and pollen development (Aya et al., 2009). DELLA, which regulates GA signaling, also integrates several other hormone signaling pathways, e.g., brassinosteroid (Bai et al., 2012), auxin (Oh et al., 2014), abscisic acid (ABA; Lim et al., 2013) and jasmonic acid (JA; Hou et al., 2013; Davière and Achard, 2016). Recently, a jasmonate responsive transporter called GTR1 was found to involve in GA transport and *gtr1* mutants were impaired in filament elongation and anther dehiscence, and were sterile (Saito et al., 2015). In *Arabidopsis*, DELLAs mediated cross-talk between GA and other signaling pathways was mediated by *O*-linked *N*-acetylglucosamine (*O*-GlcNAc) transferase (OGT) SECRET AGENT (SEC; Zentella et al., 2016). Some non-GA pathways are also associated in floral development, as is evident from phenotypic recovery of late flowers in GA-deficient mutants (Plackett et al., 2011). In an elite japonica cultivar Koshihikari, that possessed non-functional *el1* allele, production of fertile spikelets and normal grain yields similar to other elite japonica cultivars was observed (Kwon et al., 2014, 2015). This fertility suggests occurrence of either non-GA or non-DELLA GA pathways that are known to occur in tomato (Livne et al., 2015). Seedless fruit development in *Arabidopsis della* mutants is an example of parthenocarpy through non-DELLA GA signaling (Fuentes et al., 2012). The machineries of non-GA and non-DELLA pathways are, however, unknown. A rough estimate based on transcriptome analysis of tomato is that “5% of all GA regulated genes in tomato are DELLA independent” (Livne et al., 2015). Lack of understanding of non-GA and non-DELLA regulation is a major impediment in gaining complete understanding of pollen/flower development in crop plants. This is further complicated by the fact that the cross talk among different hormones regulating pollen development is still fragmented.

## Pollen Development Under Cold Stress

Plants exposed to CS show abnormal pollen development in anthers, “often producing distorted anthers or sterile pollen grains” thereby resulting in “reduced fertilization” (Nayyar et al., 2005; Oda et al., 2010; Thakur et al., 2010; Shimono et al., 2016). The most sensitive stages to CS are, “after the onset of meiosis and pollen maturation” (Boyer and McLaughlin, 2007). In rice, the time of peak tapetal activity, i.e., the transition of the tetrad to early uni-nucleate stage (young microspore, YM stage) has highest sensitivity to cold (Oliver et al., 2005) whereas in brassica, the stage is tetrad to nucleate stage (Yu et al., 2016). In tomato, most sensitive stages to CS are 11.2 and 5.6 days before anthesis (Patterson et al., 1987). CS at the time of tapetum development aborts male gamete formation and results in sterile pollen (Oliver et al., 2005; Barton et al., 2014) by perturbing carbohydrate metabolism (Oliver et al., 2005; Oda et al., 2010; De Storme and Geelen, 2014). As a whole, the temperature stress reduces pollen development, anthesis, pollen dehiscence, pollen fertility, pollination, and pollen tube growth (Li et al., 2006; Boyer and McLaughlin, 2007; Barton et al., 2014; Proud, 2015). Transcriptome analysis of meiotic anthers in chickpea revealed that genes belonging to four main categories, i.e., carbohydrate/triacylglycerol metabolism, pollen development, signal transduction, and transport were expressed differentially in tolerant anthers subjected to CS (Sharma and Nayyar, 2014). The upregulation of all but one pollen development genes in anthers of cold treated plants compared to untreated control (Sharma and Nayyar, 2014) coupled with their role in microspore/pollen growth, such as “tetrad separation, pollen expansion, increased vascular transport, fatty acid transport, pollen maturation, pollen exine formation, pollen tube growth, fertility, and pollen development (Chen et al., 2003; Hruba et al., 2005; Mashiguchi et al., 2009; Ogawa et al., 2009; Quilichini et al., 2010)” indicated that the pollen development machinery in tolerant plants remained operative even under CS. The second unique finding of the study was upregulation of all differentially expressed carbohydrate and triacylglycerol metabolism genes (Sharma and Nayyar, 2014), thereby suggesting that cold-tolerant chickpea plants produced viable pollen under CS by maintaining pollen development as well as carbohydrate/triacylglycerol metabolic pathways (Sharma, 2014; Sharma and Nayyar, 2014).

A comparison of the effect of low temperature (LT) and high temperature (HT) on pollen development shows that while the HT induces premature degradation of the tapetum at early uninuclear microspore stage (Ku et al., 2003), LT does not show tapetum degradation, instead it induces hypertrophy such as abnormal expansion (Oda et al., 2010; De Storme and Geelen, 2014) and ectopic persistence till pollen maturation thereby causing pollen sterility (Li et al., 2006). Morphological aberrations in rice tapetum subjected to LT include abnormal vacuolization, reduced dividing capacity and hypertrophy (Mamun et al., 2006). The effect of LT on pollen development is similar among mono- and dicots as is evident from cold induced abnormality and pollen sterility in chickpea, soybean, and capsicum (Mercado et al., 1997; Nayyar et al., 2005; Ohnishi et al., 2010). In comparison to this, water deficit (4 days without watering) at meiosis in wheat does not affect meiotic cell division, but induces premature spore degeneration and loss of reproductive cell orientation (Lalonde et al., 1997). The drought stressed tapetal cells persist up to 8 days after meiosis and defects in microspore polarity may be the reason for pollen sterility (De Storme and Geelen, 2014). Drought stressed tapetal cells also show abnormal vacuolization and separate from inner anther wall (Saini et al., 1984). All these studies point toward key role of tapetum in stress induced pollen sterility irrespective of the stress type or differences in morphological and cytological reaction of anthers to various types of abiotic stresses. Since, tapetum provides nutrition to developing microspores and GA signaling works primarily in the tapetum (Plackett et al., 2011), research efforts should be directed toward understanding mechanisms governing transport of carbohydrates, proteins and fatty acids, etc., via tapetum to the microspores and regulation of hormonal signaling in tapetum under normal as well as stressed conditions.

## ABA: A Potential Signal for Cold-Induced Pollen Sterility

Abscisic acid is a key endogenous messenger in plant’s responses to abiotic stresses (Baron et al., 2012; Mega et al., 2015) and is a potential signal for cold induced pollen sterility (Oliver et al., 2007). In chickpea, flowers aborting due to CS show high ABA levels indicating a possible relationship with cold-susceptibility and ABA (Thakur et al., 2010). In rice, ABA accumulates in cold-stressed anthers (3 days at 12°C) of susceptible plants leading to high level of pollen abortion (Oliver et al., 2007). Cold tolerant rice is characterized by lower endogenous ABA concentrations in anthers and the production of competent pollen (Oliver et al., 2007). ABA also plays an important role in PCD, such as the natural degradation of the tapetum (Wang et al., 1999; Ku et al., 2003). In addition to cold, higher levels of ABA also induce pollen sterility under heat and drought conditions (Tang et al., 2008; Ji et al., 2011; Bita and Gerats, 2013). This mechanism of tolerance by anthers is entirely different from that observed in leaf tissues. While, ABA in anthers induces susceptibility by inducing pollen sterility; in leaf tissues, exogenous applications of ABA or ABA mimic, a small molecule which activates downstream ABA signaling, improve abiotic stress tolerance including CS (Kumar et al., 2008; Melgoza et al., 2014; Teng et al., 2014; Cheng et al., 2016). Similarly, increased ABA levels in leaf tissues by *zeaxanthin epoxidase* (*ZEP*) and 9-*cis*-*epoxycarotenoid dioxygenase3* (*NCED*) overexpression or by *XERICO*, a gene encoding a RING-H2 zinc finger protein, in transgenic plants substantially enhance drought or salt tolerance (Espasandin et al., 2014; Estrada-Melo et al., 2015; Zhang et al., 2016). Not only CS, but HT stress was also mitigated by exogenous application of ABA (Kumar et al., 2012). The study further showed that growth reduction at HT (45/50°C; night/day) was associated with severe reduction in ABA and osmolytes.

**FIGURE 2:**
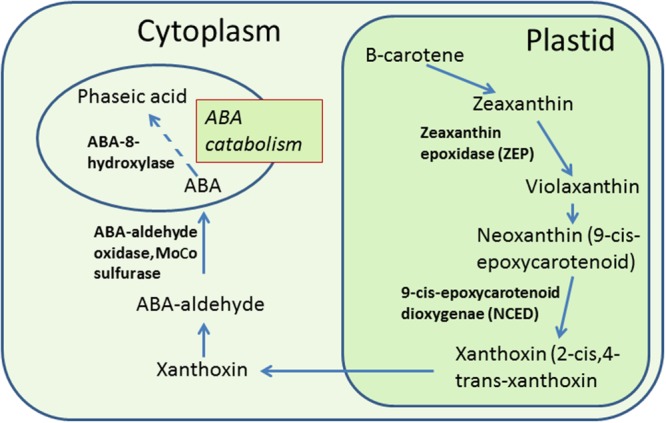
**ABA biosynthesis in plant cells and its catabolism to phaseic acid.** ABA is synthesized from carotenoids in a series of reactions in plastids and cytoplasm. In plastids, the carotenoids are converted to zeaxanthin and zeaxanthin to violaxanthin by enzyme zeaxanthin epoxidase (ZEP). Violaxanthin produces neoxanthin (9-*cis*-epoxycarotenoid) which is converted to xanthoxin (2-*cis*,4-*trans*-xanthoxin) by the oxidative cleavage of neoxanthin by the enzyme 9-*cis* epoxycarotenoid dioxygenase (NCED; Schwartz et al., 1997; see review by Seo and Koshiba, 2002). Xanthoxin is transported to the cytoplasm where it is converted to ABA by a two-step reaction. ABA is catabolized in cytoplasm to form phaseic acid. Enzyme names are shown in bold. Dotted lines indicate more than one reaction.

**FIGURE 3:**
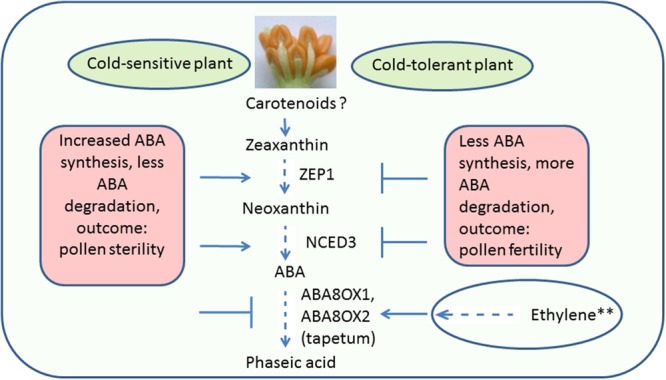
**Pathway for ABA accumulation and catabolism in anthers under cold stress.** Less amount of ABA accumulated in cold-stressed cold-tolerant anthers compared to susceptible ones, owing to reduced synthesis and increased degradation of ABA. Though, no evidence so far in anthers, ethylene, in plant leaves, positively regulate synthesis of ABA8ox1, 2, and 3, the enzymes required for ABA catabolism. Dotted lines indicate several chemical reactions; the ? Indicates absence of knowledge in anthers; the ^∗∗^ indicates evidence from leaves; the arrows show the increased and the blocked lines the decreased expression. ABA, abscisic acid; ZEP1, zeaxanthin epoxidase 1; NCED3, 9-*cis*-epoxycarotenoid dioxygenase3; ABA8ox1, ABA8′-hydroxylase 1; ABA8ox2, ABA8′-hydroxylase 2.

Abscisic acid in plant cells is synthesized from carotenoids through a series of biochemical reactions (see **Figure 2**). Upon CS, ABA levels increase in anthers as well as leaves of treated rice. The question, whether ABA is transported to anthers from leaves or is synthesized within anthers, was addressed by Oliver et al. (2007). It was found that ABA to stressed anthers is not transported from leaves, but synthesized with in anthers. Two circumstantial evidences prove this. Firstly, ABA increases in leaves regardless of pollen developmental stage, however, an increase in anthers occurs only at the YM stage, i.e., the stage of greatest cold sensitivity (Oliver et al., 2007). Secondly, ABA accumulates earlier in anthers than in leaves (Oliver et al., 2007). Increase in ABA in anthers of cold-susceptible rice results from the increased expression of two ABA biosynthetic genes named as *ZEP* (*OSZEP1* in rice) that converts zeaxanthin to violaxanthin (Oliver et al., 2007) and *NCED3* (*OSNCED3* in rice) that convert neoxanthin to xanthoxin (Oliver et al., 2007; Ji et al., 2011; **Figure 3**). The tolerant plants, on the other hand, have low expression of *OSZEP1* and *OSNCED3* compared to susceptible ones and consequently low ABA (Oliver et al., 2007; Ji et al., 2011). There is also another *NCED* gene in rice, its expression, however, does not change under CS. *OSNCED3* expresses in the xylem tissue and guard cells of stomata on the anther connective tissue in cold-stressed rice indicating that ABA biosynthesis occurs in anther vascular parenchyma cells (Ji et al., 2011). Cold tolerant plants further reduce ABA levels by increasing catabolism of ABA via C-8′ hydroxylation pathway (Nambara and Marion-Poll, 2005; **Figure 3**) thereby resulting in further reduction of ABA in anthers (Oliver et al., 2007). The expression of the ABA-8-hydroxylase genes (*ABA8ox1* and *ABA8ox2*) that convert ABA to phaseic acid was higher in tolerant rice, compared to the susceptible one (Oliver et al., 2007; Ji et al., 2011; **Figure 3**). To confirm role of ABA-hydroxylases in controlling ABA levels and inducing cold tolerance, the transgenic rice plants overexpressing wheat *TaABA8′OH1* either in tapetum or in other parts of anthers were generated (Ji et al., 2011). Overexpression of *TaABA8′OH1* in anther parts other than tapetum did not restore fertility whereas overexpression in tapetum did so thereby inducing cold-tolerance. Overexpression of *ABA8′OH1* in tapetum also resulted in lower levels of ABA and maintained *INV4* [cell wall invertase (CWIN) gene] expression (Ji et al., 2011). It has already been established that the tapetum is one of the sites of CWIN expression in anthers (Koonjul et al., 2005; Oliver et al., 2005). Clearly, low ABA and adequate CWIN activity within tapetum is key to develop viable pollen in anthers. Since, ABA appears to be synthesized in vascular parenchyma cells of the anthers (Ji et al., 2011), it might have been transported from vascular cells to the tapetum where it suppressed *INV4* expression in susceptible genotypes thereby leading to distorted sink strength and consequently the pollen sterility. Though tolerant anthers reduce ABA levels to maintain normal pollen development, the ABA levels never reach zero primarily because normal anther development and function need some amounts of ABA (Wang et al., 1999). The requirement of baseline ABA levels in anthers is similar to vegetative tissues which also need some ABA for normal growth and development (Mega et al., 2015). It has been established that non-stressed plant cells store ABA in inactive form [ABA glucosyl esters (ABA-GE)] in vacuoles and under osmotic stress, the ABA-GEs are acted upon by β-glucosidases leading to release of ABA (Xu et al., 2012). Inactive form of ABA, i.e., ABA-GE is formed by conjugation of ABA with glucose with the aid of enzyme ABA-glycosyltransferase (Lee et al., 2006; Priest et al., 2006). Since, anthers contain glucose as well as ABA, it would be interesting to explore storage of ABA as ABA-GE in vacuoles of anther cells and its immediate release from ABA-GE when anthers face CS.

Like anthers, increased expression of ABA biosynthesis and catabolism genes and lower expression of genes involved in ABA transport and homeostasis was also observed in *Arabidopsis* inflorescence meristems exposed to CS (Baron et al., 2012). Increased catabolism of ABA to phaseic acid by overexpression of *ABA*-8-*hydroxylase* gene, *OsABA8ox1*, was also observed in submerged rice (Saika et al., 2007). In submerged rice, ethylene (ET) appears to control expression of *ABA-8-hydroxylase* as treatment of aerobic seedlings with ET and its precursor, 1-aminocyclopropane-1-carboxylate (ACC), rapidly induced the expression of *OsABA8ox1* (Saika et al., 2007). On the other hand, inhibition of ET action by 1-methylcyclopropene partially suppressed induction of OsABA8ox1 expression under submergence (Saika et al., 2007).

Abscisic acid interferes with tapetum PCD (Wang et al., 1999; Ku et al., 2003; Parish et al., 2012); probably in coordination with other hormones. There is no report of cross-talk of ABA with other growth regulators in anthers, in somatic cells, indole-3-acetic acid (IAA) cross-talks with ABA. The cross-talk is mediated by a GH3 family gene, *osGH3-2*, encoding an enzyme that catalyzes conjugation of IAA to amino acids (Du et al., 2012). Rice lines overexpressing this gene showed reduced ABA and free IAA (Du et al., 2012). Under CS, the *osGH3-2* was suppressed (Du et al., 2012), thereby indicating its probable role in ABA accumulation under CS. The role of another hormone, i.e., ET in PCD leading to senescence is well documented. It is suspected that the interaction of ABA with ET causes senescence (Dolferus, 2014). The role of ET in senescence PCD and its interaction with ABA renders ET as one of the molecules whose role should be investigated in tapetum PCD as well as anther development under CS. ABA regulates negatively the ET production through ABI4-mediated transcriptional suppression of ET biosynthesis genes where ABI4 binds directly to their promoters (Dong et al., 2016). Interaction of ABA and JA in response to biotic or abiotic stresses has also been observed (Kazan and Manners, 2008). A basic helix-loop-helix–type TF, ABA-INDUCIBLE BHLH-TYPE TF/JA-ASSOCIATED MYC2-LIKE1 (JAM1), acts as a transcriptional repressor and negative regulator of JA signaling (Nakata et al., 2013) and positive regulator of ABA signaling (Li et al., 2007). Contrasting action of JAM1 on JA and ABA biosynthesis may fine tune abiotic stress responses of plants. The cross talk of ABA and GA is also well known and is discussed along with GA in the succeeding section. In addition to ABA mediated abiotic stress responses in plants, ABA independent signaling in response to osmotic stress also exists (Yoshida et al., 2014).

## ABA Signaling Regulates Sugar Metabolism and Transport

Growth of male reproductive organs of plants requires adequate amounts of sucrose which is transported to anthers from photosynthetically active cells. During early anther development in rice, lemma and palea act as sink tissues partitioning sucrose/carbohydrates from leaf/stem tissues (Zhang et al., 2010). During later stages (anther stages 9–13), lemma and palea act as source to assimilate sucrose and hexoses to anthers (Zhang et al., 2010). In a feeding assay, (^14^C) sucrose was partitioned to anthers from lemma/palea in 12 h (Zhang et al., 2010). Cells of outer anther wall cell layers and inter-connective tissues have cytoplasmic connections called plasmodesmata through which assimilate can pass to innermost cells of the middle wall layer, via the symplastic pathway (Clément and Audran, 1995). Plasmodesmata do not occur between the middle wall layer and the tapetum cells. Hence, subsequent transport of sugars, from the middle wall layer to tapetum, occurs via the complex apoplastic pathway involving enzymes such as invertases (e.g., INV4) and monosaccharide transporters (e.g., MST8; Oliver et al., 2005, 2007; Mamun et al., 2006). In sink cells, sucrose is degraded into hexoses (fructose and glucose) or their derivatives for metabolic and biosynthetic activities by CWIN (Ruan et al., 2010). CWIN are also the main components of sucrose phloem unloading pathway (Ranwala and Miller, 1998). The monosaccharides glucose and fructose are taken up by the sink tissues such as anthers through MSTs (Zhang et al., 2006). The co-expression of *CWIN* and *MST* has been observed in many systems (Roitsch and González, 2004; Zhang et al., 2010). CWIN is critical for anther development. For example, silencing of a tomato *CWIN* (*Lin5*) reduced pollen viability and pollen elongation (Zanor et al., 2009). A R2R3 MYB TF that expresses mainly in vascular tissues and tapetum in rice regulates directly the *MST8* by binding to *MST8* promoter region (Zhang et al., 2010). In addition to this, R2R3 MYB, indirectly regulates *INV4* (Zhang et al., 2010). The mutants for R2R3 MYB lack pollen viability (Zhang et al., 2010) indicating that impaired sugar transport can lead to pollen sterility.

**FIGURE 4:**
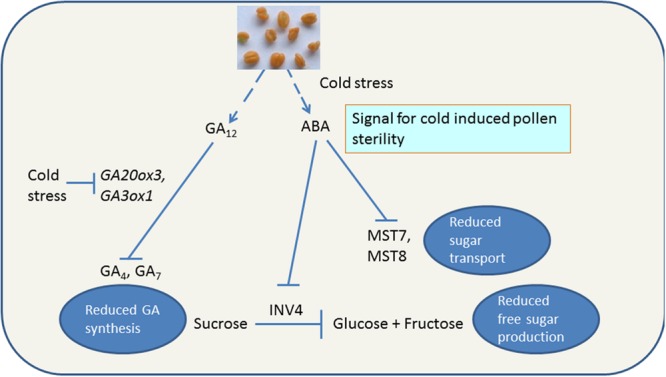
**Cold stress induced reduction in bioactive gibberellins and sugars in cold-susceptible anthers.** ABA accumulation in anthers results in pollen sterility and flower abortion by decreasing amounts of reduced free sugars. Lines with arrow show positive reaction. Blocked lines indicate inhibition of chemical reaction. Information within boxes indicate the physiological outcome of reaction. GA_12_, bioinactive GA; GA_4_, GA_7_, bioactive GAs; *GA20ox3, GA20-oxidases3*; *GA3ox1, GA3-oxidases1*; ABA, abscisic acid; MST7, monosaccharide transporter 7; MST8, monosaccharide transporter 8, INV4, cell wall invertase4.

Low temperature interrupts sugar transport and metabolism resulting in increase in accumulation of sucrose and hexose in leaves as well as floral tissues. In cold stressed rice, non-reducing sugars, e.g., sucrose accumulate in panicles of rice within 12–24 h of cold treatment (Ito, 1974), followed by tapetal hypertrophy (Ito et al., 1970). The pollen grains at YM stage of susceptible rice plants treated with LT show reduced starch accumulation and the resultant pollen developed in such plants is sterile (Oliver et al., 2005). In contrast, the cold-tolerant cultivar under LT does not accumulate excessive amounts of sucrose and the pollen remains fertile (Oliver et al., 2005; **Figure 4**). In cold-susceptible rice under LT, CWIN gene *OSINV4* (Os04g33720) was repressed, however, no repression was observed in the cold-tolerant rice (Oliver et al., 2005). *OSINV4* expresses in the tapetum at the YM stage and in the pollen grains from the early bicellular (EB) stage onward (Oliver et al., 2005). LT affects not only sucrose degradation and phloem unloading of sucrose but also monosaccharide transport. Two monosaccharide transporter genes (*OSMST8, OSMST7*) showed differential expression under LT in susceptible and tolerant rice anthers (Oliver et al., 2007; **Figure 4**). The expression of *OSMST8* under LT was higher in tolerant anthers than the susceptible ones. Another transport gene, *OSMT7* did not express in anthers under normal temperatures but at 20 times higher magnitude in tolerant anthers compared to the susceptible ones (Oliver et al., 2007). These results under LT combined with those of Zhang et al. (2010) on role of INV4 and MST8 in viable pollen development suggest that down-regulation of *OSINV4, OSMST8*, and *OSMST7* results in disruption of sucrose oﬄoading, hexose production, hexose transport, and starch formation in the pollen grains under LT (**Figure 4**).

There is a strong evidence indicating that ABA may be the potential signal for regulation of apoplastic sugar transport in anthers. Firstly, the application of ABA mimics the effect of LT in rice (Oliver et al., 2007). Secondly, ABA treatment, like LT, reduces expression of CWIN (*OSINV4*) and monosaccharide transporter genes (*OSMST8* and *OSMST7*) in susceptible rice plants (Oliver et al., 2007). Similar to rice, the ABA applications also repress the anther CWIN gene, *TaIVR1* or *INV4* in wheat (Ji et al., 2011). On the other hand, repression of invertase gene was reduced by increasing ABA catabolism (Ji et al., 2011). For example, transgenic rice lines having increased expression of wheat *TaABA8′OH1* gene under the control of the OsG6B tapetum-specific promoter, maintained the expression of invertase gene (*OSINV4*) under LT (Ji et al., 2011). The *TaABA8′OH1* over-expressing transgenic lines also have improved CS tolerance (Ji et al., 2011). In addition to ABA, glucose is another inhibitor of *CWINV* and *sucrose synthase* expression whereas GA_3_ is a positive regulator of *CWINV* and *sucrose synthase* (Zhang et al., 2014). The inhibitory effect of glucose is due to hexokinase phosphorylation of sugars and this repression is relieved by GA_3_ as has been observed in grape berries (Zhang et al., 2014). It can be concluded that GA_3_ negatively regulates the glucose signaling to maintain normal cellular sugar levels and metabolism.

Accumulation of soluble sugars following CS and sugar signaling leading to induction of cold tolerance has been studied in more detail in somatic tissues (see review by Tarkowski and Van den Ende, 2015) compared to anthers. The sugars play multifaceted roles in protecting plants from CS including stabilization of membranes under CS (Strauss and Hauser, 1986; Vereyken et al., 2001; Hincha et al., 2003); scavenging of the hydroxyl ions (Peukert et al., 2014); induction of signals to regulate stress responses of plants (Van den Ende and El-Esawe, 2014); and cross talk with other hormone pathways (Tarkowski and Van den Ende, 2015). Initial details of regulation of stress responses by sugars have been reported recently. Firstly, PtrCBF1 (C-repeat-binding factor 1), a TF and one of the central regulators of CS responses, binds directly to the promoter of *PtrBAM1*, a stress-responsive chloroplastic β-amylase-coding gene from *Poncirus trifoliate* (Peng et al., 2014). The study provided “a unique link between CBF-mediated cold responses and sugar dynamics” (Tarkowski and Van den Ende, 2015) and possibly, the partial accumulation of sugars in response to CS. Secondly, galactinol synthase, an enzyme that catalyzes first step in raffinose family oligosaccharides biosynthesis, is considered as a target gene of the CBF regulon (Taji et al., 2002). Thirdly, the DELLAs can be specifically stabilized by Sucrose (Li et al., 2014). The information from somatic tissues can be used to study differences or similarities in regulation of sugar signaling in anthers leading to viable pollen development in tolerant genotypes and sterility in susceptibility ones.

## GA Signaling Under Cold Stress

Cold stress reduces endogenous levels of the bioactive gibberellins (GAs) GA_4_ and GA_7_ in developing anthers (Sakata et al., 2014). In cold-stressed plants, the levels of precursor (GA_12_) of GA_4_ and GA_7_ were high indicating that GA_12_ was not being metabolized. The increased accumulation of GA_12_ and reduced levels of GA_4_ and GA_7_ can be attributed to lowered expression of GA biosynthesis genes, *GA20ox3* (*GA20 oxidase3*) and *GA3ox1* (*GA3 oxidase1*) under LT (**Figure 4**). Under normal conditions, expression of *GA3ox1* and *GA20ox3* increase gradually during anther development with a peak at the binucleate pollen stage (Sakata et al., 2014). Under LT, expression of *GA20ox3* was suppressed from the uninucleate to the binucleate stage whereas that of *GA3ox1* from the meiotic to binucleate stage (Sakata et al., 2014). Reduced GA synthesis but no change in GA catabolism is the outcome of LT in susceptible plants because GA catabolic genes [GA 2-oxidases (*GA2ox*)] are barely expressed in developing anthers of rice (Oda et al., 2010) with the exception of *GA2ox1*, the levels of which were repressed by cold (Sakata et al., 2014). Thus, LT severely reduces the endogenous levels of bioactive GAs through transcriptional repression of the GA biosynthetic genes *GA20ox3* and *GA3ox1*, which are strongly up-regulated during normal anther development. This is further supported by the fact that exogenous applications of GA or GA with sucrose significantly reverse the male sterility (Sakata et al., 2014). CS increases expression of the dehydration-responsive element-binding protein DREB2B and SLENDER RICE1 (SLR1)/DELLA (Sakata et al., 2014). GA-dependent GID1–SLR1 interaction also occurs *in planta* (Ueguchi-Tanaka et al., 2007; Plackett et al., 2011). Thus, there exists a possibility that LT disrupts GA-responsive pollen development through the transcriptional activation of SLR1/DELLA. This was corroborated by the fact that the mutants for GID1 (gid1) and SLR1 (slr1-d1, gain of function mutation) had fewer number of mature pollen grains under LT (Sakata et al., 2014).

GA signaling in other plant parts such as barley aleurone cells, is associated with synthesis of amylose, i.e., starch (Zentella et al., 2002). The gene α-amylase is induced by GA biosynthetic pathway TF called GAMYB whereas another regulatory protein, DELLA protein SLN1, is a repressor of GA action (Zentella et al., 2002). Furthermore, cold induced hormone, ABA, inhibits GA induced α-amylase activity. This suppression is caused by ABA-induced serine threonine protein kinase, PKABA1 (Gómez-Cadenas et al., 1999, 2001). Since, starch is important for pollen viability, GA signaling in amylose synthesis in anthers at LT should be investigated.

Gibberellic acid cross talks with other hormones to regulate development and stress responses of plants. A module of cross talk of GA with other hormones is GA repressor DELLA which interacts directly with core components of signaling cascades of several hormones (Claeys et al., 2014; Davière and Achard, 2016). During seed germination, ABA maintains dormancy whereas GA induces germination. High ABA in mature dry seed activates the TFs ABSCISIC ACID INSENSITIVE 3 (ABI3) and ABI5 both of which negatively regulate seed germination (Piskurewicz et al., 2008). The DELLA forms a complex with ABI3 and ABI5 that promotes expression of *SOMNUS* (*SOM*) which is a negative regulator of seed germination (Lim et al., 2013). Upon conditions favorable to germination, GA levels increase and degrade DELLA thereby suppressing inhibition of seed germination (Piskurewicz et al., 2008). DELLA also interferes with auxin production by interacting with auxin response factor 6 (ARF6) and inhibiting its transcription (Oh et al., 2014) whereas GA release overcomes inhibition of ARF6 (Davière and Achard, 2016). The ARF6 might have a broader role in regulating multi-hormone responses as ARF6 along with ARF8, also controls the regulation of JA-biosynthetic genes and JA biosynthesis (Nagpal et al., 2005; Tabata et al., 2010; Reeves et al., 2012). Interaction of GA and ET was observed in induction of hook curvature where GA_3_ enhanced ET- and ET INSENSITIVE3 (EIN3)-overexpression-induced hook curvature, and DELLA proteins interacted with the DNA-binding domains of EIN3/EIL1 EIN3-like and repressed EIN3/EIL1-regulated *HOOKLESS1* (*HLS1*) expression (An et al., 2012). Cross talk between JA and GA also occur during plant development and defense. GAs and JAs activate the expression of the R2R3 MYB TFs, MYB21, MYB24, and MYB57, involved in anther and stamen development (Cheng et al., 2009). JA releases the repressive activity of JASMONATE ZIM DOMAIN (JAZs) onto MYB TFs (Cheng et al., 2009; Song et al., 2013) where DELLA interact with JAZ (Hou et al., 2013) and JAZ proteins act as repressors of MYC2, a TF that regulates JA responses (Lorenzo et al., 2004).

## ET and Cold Stress

Though, no evidence exists so far, ET may be the other hormone regulating anther cold tolerance. Firstly, ET receptor genes are present in anthers. Secondly, the anther-specific expression of mutated melon ET receptor gene *Cm-ERS1/H70A* delays tapetum degeneration and causes pollen abortion (Takada et al., 2006). Thirdly, in somatic tissues, ET is a regulator of cold or freezing tolerance (Kazan, 2015). Though, role of ET in plant PCD is well documented, there is no report of involvement of ET in delay in tapetum degeneration under CS and this aspect needs investigation. ET acts as a growth inhibitor as well as growth promoter, e.g., etiolation (Dolferus, 2014). During anther development, ET appears to play a double role; at low levels it causes tapetum PCD leading to normal pollen development (Takada et al., 2006) whereas at higher levels it causes premature tapetal degeneration (Wei et al., 2013) leading to pollen sterility. Under normal male gametophyte development, two ET peaks occur in anthers; the first peak coincides with degeneration of tapetum and the middle layers of the anther wall and the second with maturation and dispersal of pollen grains (Kovaleva et al., 2011). In genetic male-sterile lines (GMS), excessively high ET levels and higher expression of ET metabolism genes coincided with the ET peak (in cotton) as well as the time of tapetal death (in petunia; Kovaleva et al., 2011; Wei et al., 2013). The anthers of GMS also had higher expression of ET-responsive TF 12 (Zhang et al., 2015). Two ET Response Factor-associated amphiphilic repression motif proteins called DAZ1 and DAZ2 are required for germ cells to enter mitosis (Borg et al., 2011). These proteins also regulate expression of genes involved in differentiation of germ-lines (Borg et al., 2014). ET also cross talks with other regulators. ET-auxin crosstalk occurs in plant roots and is necessary for root elongation (Lewis et al., 2011).

Unlike anthers, role of ET in cold tolerance or susceptibility in somatic tissues has been investigated, however, complexity of its function vis-a-vis tolerance or susceptibility is still elusive as in some cases it causes susceptibility whereas in others it causes tolerance. In *Medicago truncatula*, a negative correlation was found between ET levels and cold-tolerance (Zhao et al., 2014) whereas in tomato, tobacco (Zhang and Huang, 2010), and *Arabidopsis* (Catalá et al., 2014), the correlation between ET and freezing-tolerance was positive. The contrasting roles of ET in regulating cold tolerance or susceptibility cannot be explained at present as mechanisms by which ET regulate plants’ responses under CS are poorly understood.

## Other Hormones and Cold Stress

Jasmonic acid regulates stamen development as plants defective in JA synthesis were also defective in stamens (Nakata et al., 2013). Mutations in JA biosynthesis genes inhibit filament elongation, delay anther dehiscence, and induce pollen sterility whereas exogenous application of JA restores fertility in JA gene mutants (Caldelari et al., 2011; Nakata et al., 2013). Auxins also regulate stamen development and these act through JA to control anther development (Nakata et al., 2013). Similar to JA mutants, *arf6-2* and *arf8-3* double mutant arrests stamen development in *Arabidopsis* (Nagpal et al., 2005). Both the ARF6 and ARF8 control the expressions of JA-biosynthetic genes and regulate JA biosynthesis in flower buds (Nagpal et al., 2005; Tabata et al., 2010). JA and ET also interact to induce pathogen defense and regulate growth (Song et al., 2014; Kim et al., 2015). The ET genes *EIN3* and *EIL1* positively regulate JA-mediated responses by physical interaction of JAZ proteins (e.g., JAZ1, 3, and 9) with EIN3 and EIL1 leading to repression of *EIN3/EIL1* transcription (Zhu et al., 2011).

Jasmonic acid in somatic tissues, is a positive regulator of cold and freezing tolerance (Kazan, 2015; Sharma and Laxmi, 2015), as evident from decreased freezing tolerance in JA biosynthesis impaired mutants and increased freezing tolerance in JA treated plants (Hu et al., 2013). JAZs physically interact and suppress INDUCER OF CBF EXPRESSION 1 (ICE1) and ICE2 (Hu et al., 2013). Similar to JA, role of auxins in cold tolerance in anthers has not been investigated so far, however, LT in somatic tissues, reduces auxin levels (Nadella et al., 2006; Zhu et al., 2015). Reduced auxins, thus induce cold tolerance; the mechanism, however, is not useful agriculturally, as low auxin levels suppress growth and hence, surely will decrease crop yields.

## Conclusion

Carbohydrate metabolism, carbohydrate transport and bioactive GAs are keys to cold tolerance by anthers. Failure of pollen development in cold-susceptible plants occurs as a result of reduced degradation of sucrose to hexoses owing to reduced invertase, reduced monosaccharide transport by impaired sugar transporter activity, decreased starch accumulation via some unknown mechanisms and reduced amounts of bioactive GAs. Sucrose degradation and transport is regulated by ABA, which accumulates in higher amounts in susceptible anthers under stress. Unlike carbohydrates, there is no evidence at present suggesting that ABA is linked to lower GA synthesis in susceptible anthers or GA is linked to distorted carbohydrate metabolism including starch accumulation. Indirect evidences in leaves indicate that complex interactions involving ABA, GA, and sugar signaling may exist in anthers. ET, JA, and auxins also regulate stamen development and these are the other potential but least studied pathways involved in anther development under CS. Crosstalk of several hormones in somatic tissues is well documented, no such study has, however, been conducted in anthers so far.

In contrast to susceptibility, ability of anthers to develop viable pollen under CS depends upon the genotype’s capability to accumulate lower ABA and maintain adequate pool of bioactive GAs. Such “cold-tolerant” genotypes, not only synthesize lower amounts of ABA but also have increased ABA catabolism by enhanced expression of ABA hydroxylation genes. For bioactive GAs, low accumulation in susceptible genotypes occurs due to repression of biosynthetic genes and not due to catabolism of bioactive GAs. The tolerant anthers maintain normal carbohydrate metabolism including adequate deposition of starch in anther grains. The mechanisms of adequate starch accumulation in cold-tolerant plants and low starch accumulation in susceptible ones are not known. Based on initial indications, the role of ABA and GA pathways is suspected, hence, there is a need to investigate the role of these hormones in inhibition of starch biosynthesis in cold stressed anthers. Anthers, being a tissue with highly specific function in plant reproduction, might employ cold-tolerance mechanisms dissimilar to somatic tissue as is evident in case of ABA where increased levels in somatic parts induced cold-tolerance but in anthers, higher ABA caused pollen sterility, i.e., cold-susceptibility.

To understand cold tolerance mechanisms in anthers, future research should be focussed on GA signaling in starch synthesis in anthers, effect of ABA on inhibition of GA induced α-amylase activity and starch biosynthesis and effect of ABA on inhibition of tapetum PCD. In addition, the roles of ET, auxins, JA, sugar and Ca^2+^ signaling in anther cold-tolerance leading to viable pollen development including crosstalk of these pathways should also be investigated.

## Author Contribitions

All authors listed, have made substantial, direct and intellectual contribution to the work, and approved it for publication.

## Conflict of Interest Statement

The authors declare that the research was conducted in the absence of any commercial or financial relationships that could be construed as a potential conflict of interest.

The reviewer EFC and handling Editor declared their shared affiliation, and the handling Editor states that the process nevertheless met the standards of a fair and objective review.

## References

[B1] An F. Zhang X. Zhu Z. Ji Y. He W. Jiang Z. (2012). Coordinated regulation of apical hook development by gibberellins and ethylene in etiolated *Arabidopsis* seedlings. *Cell Res* 22 915–927. 10.1038/cr.2012.29 22349459 PMC3343656

[B2] Aya K. Suzuki G. Suwabe K. Hobo T. Takahashi H. (2011). Comprehensive network analysis of anther-expressed genes in rice by the combination of 33 laser microdissection and 143 spatiotemporal microarrays. *PLoS ONE* 6:e26162 10.1371/journal.pone.0026162 PMC3202526 22046259

[B3] Aya K. Ueguchi-Tanaka M. Kondo M. Hamada K. Yano K. Nishimura M. (2009). Gibberellin modulates anther development in rice via the transcriptional regulation of GAMYB. *Plant Cell* 21 1453–1472. 10.1105/tpc.108.062935 19454733 PMC2700530

[B4] Bai M. Y. Shang J. X. Oh E. Fan M. Bai Y. Zentella R. (2012). Brassinosteroid, gibberellin and phytochrome impinge on a common transcription module in *Arabidopsis*. *Nat. Cell Biol.* 14 810–817. 10.1038/ncb2546 22820377 PMC3606816

[B5] Baron K. N. Schroeder D. F. Stasolla C. (2012). Transcriptional response of abscisic acid (ABA) metabolism and transport to cold and heat stress applied at the reproductive stage of development in *Arabidopsis thaliana*. *Plant Sci.* 188–189, 48–59. 10.1016/j.plantsci.2012.03.001 22525244

[B6] Barton D. A. Cantrill L. C. Law A. M. Phillips C. G. Sutton B. G. Overall R. L. (2014). Chilling to zero degrees disrupts pollen formation but not meiotic microtubule arrays in *Triticum aestivum* L. *Plant Cell Environ.* 37 2781–2794. 10.1111/pce.12358 24762030

[B7] Bita C. E. Gerats T. (2013). Plant tolerance to high temperature in a changing environment: scientific fundamentals and production of heat stress-tolerant crops. *Front. Plant Sci.* 4:273 10.3389/fpls.2013.00273 PMC3728475 23914193

[B8] Borg M. Brownfield L. Khatab H. Sidorova A. Lingaya M. Twell D. (2011). The R2R3 MYB transcription factor DUO1 activates a male germline-specific regulon essential for sperm cell differentiation in *Arabidopsis*. *Plant Cell* 23 534–549. 10.1105/tpc.110.081059 21285328 PMC3077786

[B9] Borg M. Rutley N. Kagale S. Hamamura Y. Gherghinoiu M. Kumar S. (2014). An EAR-dependent regulatory module promotes male germ cell division and sperm fertility in *Arabidopsis*. *Plant Cell* 26 2098–2113. 10.1105/tpc.114.124743 24876252 PMC4079371

[B10] Boyer J. S. McLaughlin J. E. (2007). Functional reversion to identify controlling genes in multigenic responses: analysis of floral abortion. *J. Exp. Bot.* 58 267–277. 10.1093/jxb/erl177 17105969

[B11] Caldelari D. Wang G. Farmer E. E. Dong X. (2011). *Arabidopsis* lox3 lox4 double mutants are male sterile and defective in global proliferative arrest. *Plant Mol. Biol.* 75 25–33. 10.1007/s11103-010-9701-9 21052784

[B12] Catalá R. López-Cobollo R. Castellano M. M. Angosto T. Alonso J. M. Ecker J. R. (2014). The *Arabidopsis* 14-3-3 protein RARE COLD INDUCIBLE 1A links low-temperature response and ethylene biosynthesis to regulate freezing tolerance and cold acclimation. *Plant Cell* 26 3326–3342. 10.1105/tpc.114.127605 25122152 PMC4371832

[B13] Chang F. Wang Y. Wang S. Ma H. (2011). Molecular control of microsporogenesis in *Arabidopsis*. *Curr. Opin. Plant Biol.* 14 66–73. 10.1016/j.pbi.2010.11.001 21145279

[B14] Chen X. Goodwin S. M. Boroff V. L. Liu X. Jenks M. A. (2003). Cloning and characterization of the WAX2 gene of *Arabidopsis* involved in cuticle membrane and wax production. *Plant Cell* 15 1170–1185. 10.1105/tpc.010926 12724542 PMC153724

[B15] Cheng H. Song S. Xiao L. Soo H. M. Cheng Z. Xie D. (2009). Gibberellin acts through jasmonate to control the expression of MYB21, MYB24, and MYB57 to promote stamen filament growth in *Arabidopsis*. *PLoS Genet.* 5:e1000440 10.1371/journal.pgen.1000440 PMC2654962 19325888

[B16] Cheng Z. Jin R. Cao M. Liu X. Chan Z. (2016). Exogenous application of ABA mimic 1 (AM1) improves cold stress tolerance in bermudagrass (*Cynodon dactylon*). *Plant Cell Tissue Organ Cult.* 10.1007/s11240-016-0941-5

[B17] Claeys H. De Bodt S. Inzé D. (2014). Gibberellins and DELLAs: central nodes in growth regulatory networks. *Trends Plant Sci.* 19 231–239. 10.1016/j.tplants.2013.10.001 24182663

[B18] Cleément C. Audran J. C. (1995). Anther wall layers control pollen sugar nutrition in Lilium. *Protoplasma* 187 172–181. 10.1007/BF01280246

[B19] Dai C. Xue W. (2010). Rice early flowering1, a CKI, phosphorylates DELLA protein SLR1 to negatively regulate gibberellin. *EMBO J.* 29 1916–1927. 10.1038/emboj.2010.75 20400938 PMC2885930

[B20] Davière J. M. Achard P. (2016). A pivotal role of DELLAs in regulating multiple hormone signals. *Mol. Plant* 9 10–20. 10.1016/j.molp.2015.09.011 26415696

[B21] De Storme N. Geelen D. (2014). Callose homeostasis at plasmodesmata: molecular regulators and developmental relevance. *Front. Plant Sci.* 5:138 10.3389/fpls.2014.00138 PMC4001042 24795733

[B22] Dolferus R. (2014). To grow or not to grow: a stressful decision for plants. *Plant Sci.* 229 247–261. 10.1016/j.plantsci.2014.10.002 25443851

[B23] Dong Z. Yu Y. Li S. Wang J. Tang S. Huang R. (2016). Abscisic acid antagonizes ethylene production through the ABI4-mediated transcriptional repression of ACS4 and ACS8 in *Arabidopsis*. *Mol. Plant* 9 126–135. 10.1016/j.molp.2015.09.007 26410794

[B24] Du H. Wu N. Fu J. Wang S. Li X. Xiao J. (2012). GH3 family member, OsGH3-2, modulates auxin and abscisic acid levels and differentially affects drought and cold tolerance in rice. *J. Exp. Bot.* 63 6467–6480. 10.1093/jxb/ers300 23112280 PMC3504496

[B25] Espasandin F. D. Maiale S. J. Calzadilla P. Ruiz O. A. Sansberro P. A. (2014). Transcriptional regulation of 9-cis-epoxycarotenoid dioxygenase (NCED) gene by putrescine accumulation positively modulates ABA synthesis and drought tolerance in *Lotus tenuis* plants. *Plant Physiol. Biochem.* 76 29–35. 10.1016/j.plaphy.2013.12.018 24448322

[B26] Estrada-Melo A. C. Chao M. S. R. Jiang C. Z. (2015). Overexpression of an ABA biosynthesis gene using a stress-inducible promoter enhances drought resistance in petunia. *Horticult. Res.* 2:15013 10.1038/hortres.2015.13 PMC4595983 26504568

[B27] Fuentes S. Ljung K. Sorefan K. Alvey E. Harberd N. P. Østergaard L. (2012). Fruit growth in *Arabidopsis occurs* via DELLA-dependent and DELLA-independent gibberellin responses. *Plant Cell* 24 3982–3996. 10.1105/tpc.112.103192 23064323 PMC3517231

[B28] Gómez J. F. Talle B. Wilson Z. A. (2015). Anther and pollen development: a conserved developmental pathway. *J. Integr. Plant Biol.* 57 876–891. 10.1111/jipb.12425 26310290 PMC4794635

[B29] Gómez-Cadenas A. Verhey S. D. Holappa L. D. Shen Q. Ho T. H. Walker-Simmons M. K. (1999). An abscisic acid induced protein kinase, PKABA1, mediates abscisic acid-suppressed gene expression in barley aleurone layers. *Proc. Natl. Acad. Sci. U.S.A.* 96 1767–1772. 10.1073/pnas.96.4.1767 9990099 PMC15589

[B30] Gómez-Cadenas A. Zentella R. Walker-Simmons M. K. Ho T. H. (2001). Gibberellin/abscisic acid antagonism in barley aleurone cells: site of action of the protein kinase PKABA1 in relation to gibberellin signaling molecules. *Plant Cell* 13 667–679. 10.2307/3871414 11251104 PMC135510

[B31] Hincha D. K. Zuther E. Heyer A. G. (2003). The preservation of liposomes by raffinose family oligosaccharides during drying is mediated by effects on fusion and lipid phase transitions. *Biochim. Biophys. Acta* 1612 172–177. 10.1016/S0005-2736(03)00116-0 12787935

[B32] Hord C. L. Chen C. Deyoung B. J. Clark S. E. Ma H. (2006). The BAM1/BAM2 receptor-like kinases are important regulators of *Arabidopsis* early anther development. *Plant Cell* 18 1667–1680. 10.1105/tpc.105.036871 16751349 PMC1488923

[B33] Hou X. Ding L. Yu H. (2013). Crosstalk between GA and JA signaling mediates plant growth and defense. *Plant Cell Rep.* 32 1067–1074. 10.1007/s00299-013-1423-4 23525761

[B34] Hruba P. Honys D. Twell D. Capkova V. Tupy J. (2005). Expression of β-galactosidase and β-xylosidase genes during microspore and pollen development. *Planta* 220 931–940. 10.1007/s00425-004-1409-0 15517348

[B35] Hu Y. Jiang L. Wang F. Yu D. (2013). Jasmonate regulates the inducer of CBF expression–C-repeat binding factor/DRE binding factor1 cascade and freezing tolerance in *Arabidopsis*. *Plant Cell* 25 2907–2924. 10.1105/tpc.113.112631 23933884 PMC3784588

[B36] Ito N. (1974). “Change of carbohydrates in anthers cooled at the young microspore stage,” in *Proceeding of the Crop Science Society* Tokyo 179–180.

[B37] Ito N. Hayase H. Satake T. Nishiyama I. (1970). “Male sterility caused by cooling treatment at the meiotic stage in rice plants. III. Male abnormalities at anthesis,” in *Proceeding of Crop Science Society* Tokyo 60–64.

[B38] Ito T. Wellmer F. Yu H. Das P. Ito N. Alves-Ferreira M. (2004). The homeotic protein AGAMOUS controls microsporogenesis by regulation of SPOROCYTELESS. *Nature* 430 356–360. 10.1038/nature02733 15254538

[B39] Ji X. M. Dong B. D. Shiran B. Talbot M. J. Edlington J. E. Hughes T. (2011). Control of abscisic acid catabolism and abscisic acid homeostasis is important for reproductive stage stress tolerance in cereals. *Plant Physiol.* 156 647–662. 10.1104/pp.111.176164 21502188 PMC3177265

[B40] Kazan K. (2015). Diverse roles of jasmonates and ethylene in abiotic stress tolerance. *Trends Plant Sci.* 20 219–229. 10.1016/j.tplants.2015.02.001 25731753

[B41] Kazan K. Manners J. M. (2008). Jasmonate signaling: toward an integrated view. *Plant Physiol.* 146 1459–1468. 10.1104/pp.107.115717 18390489 PMC2287326

[B42] Kelliher T. Egger R. L. Zhang H. Walbot V. (2014). Unresolved issues in pre-meiotic anther development. *Front. Plant Sci.* 5:347 10.3389/fpls.2014.00347 PMC4104404 25101101

[B43] Kim J. Chang C. Tucker M. L. (2015). To grow old: regulatory role of ethylene and jasmonic acid in senescence. *Front. Plant Sci.* 6:20 10.3389/fpls.2015.00020 PMC4310285 25688252

[B44] Koonjul P. K. Minhas J. S. Nunes C. Sheoran I. S. Saini H. S. (2005). Selective transcriptional down-regulation of anther invertases precedes the failure of pollen development in water-stressed wheat. *J. Exp. Bot.* 56 179–190. 15533880 10.1093/jxb/eri018

[B45] Kovaleva L. V. Alla D. Alexander V. Viktor R. (2011). Ethylene is involved in the control of male gametophyte development and germination in Petunia. *J. Plant Growth Regulat.* 30 64–73. 10.1007/s00344-010-9168-6

[B46] Ku S. Yoon H. Suh H. S. Chung Y. Y. (2003). Male-sterility of thermo-sensitive genic male-sterile rice is associated with premature programmed cell death of the tapetum. *Planta* 217 559–565. 10.1007/s00425-003-1030-7 12692728

[B47] Kumar S. Kaur G. Nayyar H. (2008). Exogenous application of abscisic acid improves cold tolerance in chickpea (*Cicer arietinum* L.). *J. Agron. Crop Sci.* 194 449–456.

[B48] Kumar S. Kaushal N. Nayyar H. Gaur P. (2012). Abscisic acid induces heat tolerance in chickpea (*Cicer arietinum* L.) seedlings by facilitated accumulation of osmoprotectants. *Acta Physiol. Plant.* 34 1651–1658. 10.1007/s11738-012-0959-1

[B49] Kwon C. T. Kim S. H. Kim D. Paek N. C. (2015). The rice floral repressor early flowering1 affects spikelet fertility by modulating gibberellin signaling. *Rice* 8 1–11. 10.1186/s12284-015-0058-1 PMC4584262 26202549

[B50] Kwon C. T. Yoo S. C. Koo B. H. Cho S. H. Park J. W. Zhang Z. (2014). Natural variation in Early flowering1 contributes to early flowering in japonica rice under long days. *Plant Cell Environ.* 37 101–112. 10.1111/pce.12134 23668360

[B51] Lalonde S. Beebe D. U. Saini H. S. (1997). Early signs of disruption of wheat anther development associated with the induction of male sterility by meiotic-stage water deficit. *Sex. Plant Reproduct.* 10 40–48. 10.1007/s004970050066

[B52] Lee K. H. Piao H. L. Kim H. Y. Choi S. M. Jiang F. Hartung W. (2006). Activation of glucosidase via stress-induced polymerization rapidly increases active pools of abscisic acid. *Cell* 126 1109–1120. 10.1016/j.cell.2006.07.034 16990135

[B53] Lewis D. R. Negi S. Sukumar P. Muday G. K. (2011). Ethylene inhibits lateral root development, increases IAA transport and expression of PIN3 and PIN7 auxin eﬄux carriers. *Development* 138 3485–3495. 10.1242/dev.065102 21771812

[B54] Li M. Cha D. J. Lai Y. Villaruz A. E. Sturdevant D. E. Otto M. (2007). The antimicrobial peptide-sensing system aps of *Staphylococcus aureus*. *Mol. Microbiol.* 66 1136–1147. 10.1111/j.1365-2958.2007.05986.x 17961141

[B55] Li N. Zhang D. S. Liu H. S. Yin C. S. Li X. X. Liang W. (2006). The rice tapetum degeneration retardation gene is required for tapetum degradation and anther development. *Plant Cell* 18 2999–3014. 10.1105/tpc.106.044107 17138695 PMC1693939

[B56] Li Y. Van den Ende W. Rolland F. (2014). Sucrose induction of anthocyanin biosynthesis is mediated by DELLA. *Mol. Plant* 7 570–572. 10.1093/mp/sst161 24243681

[B57] Lim S. Park J. Lee N. Jeong J. Toh S. Watanabe A. (2013). ABA-INSENSITIVE_3_, ABA-INSENSITIVE_5_, and DELLAs interact to activate the expression of *SOMNUS* and other high-temperature-inducible genes in imbibed seeds in *Arabidopsis*. *Plant Cell* 25 4863–4878. 10.1105/tpc.113.118604 24326588 PMC3903992

[B58] Liu J. Zhang Y. Qin G. Tsuge T. Sakaguchi N. (2008). Targeted degradation of the cyclin-dependent kinase inhibitor ICK4/KRP6 by RING-type E3 ligases is essential for mitotic cell cycle progression during *Arabidopsis* gametogenesis. *Plant Cell* 20 1538–1554. 10.1105/tpc.108.059741 18552199 PMC2483368

[B59] Liu X. Huang J. Parameswaran S. Ito T. Seubert B. Auer M. (2009). The SPOROCYTELESS/NOZZLE gene is involved in controlling stamen identity in *Arabidopsis*. *Plant Physiol.* 151 1401–1411. 10.1104/pp.109.145896 19726570 PMC2773108

[B60] Livne S. Lor V. S. Nir I. Eliaz N. Aharoni A. Olszewski N. E. (2015). Uncovering della-independent gibberellin responses by characterizing new tomato procera mutants. *Plant Cell* 27 1579–1594. 10.1105/tpc.114.132795 26036254 PMC4498196

[B61] Lorenzo O. Chico J. M. Sánchez-Serrano J. J. Solano R. (2004). JASMONATE-INSENSITIVE1 encodes a MYC transcription factor essential to discriminate between different jasmonate-regulated defense responses in *Arabidopsis*. *Plant Cell* 16 1938–1950. 10.1105/tpc.022319 15208388 PMC514172

[B62] Mamun E. A. Alfred S. Cantrill L. C. Overall R. L. Sutton B. G. (2006). Effects of chilling on male gametophyte development in rice. *Cell Biol. Int.* 30 583–591. 10.1016/j.cellbi.2006.03.004 16730464

[B63] Mashiguchi K. Asami T. Suzuki Y. (2009). Genome-wide identification, structure and expression studies, and mutant collection of 22 early nodulin-like protein genes in *Arabidopsis*. *Biosci. Biotechnol. Biochem.* 73 2452–2459. 10.1271/bbb.90407 19897921

[B64] Mega R. Meguro-Maoka A. Endo A. Shimosaka E. Murayama S. Nambara E. (2015). Sustained low abscisic acid levels increase seedling vigor under cold stress in rice (*Oryza sativa* L.). *Sci. Rep.* 5:13819 10.1038/srep13819 PMC4563555 26350634

[B65] Melgoza F. J. Kusakabe A. Nelson S. D. Melgar J. C. (2014). Exogenous applications of abscisic acid increase freeze tolerance in citrus trees. *Int. J. Fruit Sci.* 14 376–387. 10.1080/15538362.2014.899138

[B66] Mercado J. A. Mar Trigo M. Reid M. S. Valpuesta V. Quesada M. A. (1997). Effects of low temperature on pepper pollen morphology and fertility: evidence of cold induced exine alterations. *J. Horticult. Sci.* 72 317–326. 10.1080/14620316.1997.11515518

[B67] Nadella V. Shipp M. J. Muday G. K. Wyatt S. E. (2006). Evidence for altered polar and lateral auxin transport in the gravity persistent signal (gps) mutants of *Arabidopsis.* *Plant Cell Environ.* 29 682–690. 10.1111/j.1365-3040.2005.01451.x 17080617

[B68] Nagpal P. Ellis C. M. Weber H. Ploense S. E. Barkawi L. S. Guilfoyle T. J. (2005). Auxin response factors ARF6 and ARF8 promote jasmonic acid production and flower maturation. *Development* 132 4107–4118. 10.1242/dev.01955 16107481

[B69] Nakata M. Mitsuda N. Herde M. Koo A. J. Moreno J. E. Suzuki K. (2013). A bHLH-type transcription factor, ABA-INDUCIBLE BHLH-TYPE TRANSCRIPTION FACTOR/JA-ASSOCIATED MYC2-LIKE1, acts as a repressor to negatively regulate jasmonate signaling in *Arabidopsis*. *Plant Cell* 25 1641–1656. 10.1105/tpc.113.111112 23673982 PMC3694697

[B70] Nakazono M. Qiu F. Borsuk L. A. Schnable P. S. (2003). Laser-capture microdissection, a tool for the global analysis of gene expression in specific plant cell types: identification of genes expressed differentially in epidermal cells or vascular tissues of maize. *Plant Cell* 15 583–596. 10.1105/tpc.008102 12615934 PMC150015

[B71] Nambara E. Marion-Poll A. (2005). Abscisic acid biosynthesis and catabolism. *Annu. Rev. Plant Biol.* 56 165–185. 10.1146/annurev.arplant.56.032604.144046 15862093

[B72] Nayyar H. Bains T. S. Kumar S. (2005). Low temperature induced floral abortion in chickpea: relationship to abscisic acid and cryoprotectants in reproductive organs. *Environ. Exp. Bot.* 53 39–47. 10.1016/j.envexpbot.2004.02.011

[B73] Oda S. Kaneko F. Yano K. Fujioka T. Masuko H. Park J. I. (2010). Morphological and gene expression analysis under cool temperature conditions in rice anther development. *Genes Genet. Syst.* 85 107–120. 10.1266/ggs.85.107 20558897

[B74] Ogawa M. Kay P. Wilson S. Swain S. M. (2009). Arabidopsis dehiscence zone polygalacturonase1 (ADPG1), ADPG2 and QUARTET2 are polygalacturonases required for cell separation during reproductive development in *Arabidopsis*. *Plant Cell* 21 216–233. 10.1105/tpc.108.063768 19168715 PMC2648098

[B75] Oh E. Zhu J. Y. Bai M. Y. Arenhart R. A. Sun Y. Wang Z. Y. (2014). Cell elongation is regulated through a central circuit of interacting transcription factors in the *Arabidopsis hypocotyl*. *Elife* 3:e03031 10.7554/eLife.03031 PMC4075450 24867218

[B76] Ohnishi S. Miyoshi T. Shirai S. (2010). Low temperature stress at different flower developmental stages affects pollen development, pollination, and pod set in soybean. *Environ. Exp. Bot.* 69 56–62. 10.1016/j.envexpbot.2010.02.007

[B77] Oliver S. N. Dennis E. S. Dolferus R. (2007). ABA regulates apoplastic sugar transport and is a potential signal for cold-induced pollen sterility in rice. *Plant Cell Physiol.* 48 1319–1330. 10.1093/pcp/pcm100 17693452

[B78] Oliver S. N. Van Dongen J. T. Alfred S. C. Mamun E. A. Zhao X. Saini H. S. (2005). Cold-induced repression of the rice anther-specific cell wall invertase gene OSINV4 is correlated with sucrose accumulation and pollen sterility. *Plant Cell Environ.* 28 1534–1551. 10.1111/j.1365-3040.2005.01390.x

[B79] Parish R. W. Li S. F. (2010). Death of a tapetum: a programme of developmental altruism. *Plant Sci.* 178 73–89. 10.1016/j.plantsci.2009.11.001

[B80] Parish R. W. Phan H. A. Iacuone S. Li S. F. (2012). Tapetal development and abiotic stress: a centre of vulnerability. *Funct. Plant Biol.* 39 553–559. 10.1071/FP12090 32480807

[B81] Patterson B. D. Mutton L. Paull R. E. Nguyen V. Q. (1987). Tomato pollen development: stages sensitive to chilling and a natural environment for the selection of resistant genotypes. *Plant Cell Environ.* 10 363–368. 10.1111/1365-3040.ep11603604

[B82] Peng T. Zhu X. Duan N. Liu J. H. (2014). PtrBAM1, a β-amylase-coding gene of *Poncirus trifoliata*, is a CBF regulon member with function in cold tolerance by modulating soluble sugar levels. *Plant Cell Environ.* 37 2754–2767. 10.1111/pce.12384 24905016

[B83] Peukert M. Thiel J. Peshev D. Weschke W. Van den Ende W. Mock H. P. (2014). Spatio-temporal dynamics of fructan metabolism in developing barley grains. *Plant Cell* 9 3728–3744. 10.1105/tpc.114.130211 25271242 PMC4213166

[B84] Piskurewicz U. Jikumaru Y. Kinoshita N. Nambara E. Kamiya Y. Lopez-Molina L. (2008). The gibberellic acid signaling repressor RGL2 inhibits *Arabidopsis* seed germination by stimulating abscisic acid synthesis and ABI5 activity. *Plant Cell* 20 2729–2745. 10.1105/tpc.108.061515 18941053 PMC2590721

[B85] Plackett A. R. Ferguson A. C. Powers S. J. Wanchoo-Kohli A. Phillips A. L. Wilson Z. A. (2014). DELLA activity is required for successful pollen development in the Columbia ecotype of *Arabidopsis*. *New Phytol.* 201 825–836. 10.1111/nph.12571 24400898 PMC4291109

[B86] Plackett A. R. G. Thomas S. G. Wilson Z. A. Hedden P. (2011). Gibberellin control of stamen development: a fertile field. *Trends Plant Sci.* 16 568–578. 10.1016/j.tplants.2011.06.007 21824801

[B87] Priest D. M. Ambrose S. J. Vaistij F. E. Elias L. Higgins G. S. Ross A. R. (2006). Use of the glucosyltransferase UGT71B6 to disturb abscisic acid homeostasis in *Arabidopsis thaliana.* *Plant J.* 46 492–502. 10.1111/j.1365-313X.2006.02701.x 16623908

[B88] Proud C. (2015). “Cold temperature under aerobic conditions increases spikelet sterility in rice (*Oryza sativa* L.),” in *Proceedings of the 17th ASA Conference, 20 – 24 September* Hobart Available at: www.agronomy2015.com.au

[B89] Qin Z. Zhang X. Zhang X. Xin W. Li J. Hu Y. (2014). The *Arabidopsis* transcription factor IIB-related protein BRP4 is involved in the regulation of mitotic cell-cycle progression during male gametogenesis. *J. Exp. Bot.* 65 2521–2531. 10.1093/jxb/eru140 24723406 PMC4036515

[B90] Quilichini T. D. Douglas C. J. Samuels A. L. (2014). New views of tapetum ultrastructure and pollen exine development in *Arabidopsis thaliana*. *Ann. Bot.* 114 1189–1201. 10.1093/aob/mcu042 24723448 PMC4195548

[B91] Quilichini T. D. Friedmann M. C. Samuels A. L. Douglas C. J. (2010). ATP-binding cassette transporter G26 is required for male fertility and pollen exine formation in *Arabidopsis*. *Plant Physiol.* 154 678–690. 10.1104/pp.110.161968 20732973 PMC2949020

[B92] Ranwala A. P. Miller W. B. (1998). Sucrose-cleaving enzymes and carbohydrate pools in *Lilium longiflorum* floral organs. *Plant Physiol.* 103 541–550. 10.1034/j.1399-3054.1998.1030413.x

[B93] Reeves P. H. Ellis C. M. Ploense S. E. Wu M. F. Yadav V. Tholl D. (2012). A regulatory network for coordinated flower maturation. *PLoS Genet.* 8:e1002506 10.1371/journal.pgen.1002506 PMC3276552 22346763

[B94] Roitsch T. González M. C. (2004). Function and regulation of plant invertases: sweet sensations. *Trends Plant Sci.* 9 606–613. 10.1016/j.tplants.2004.10.009 15564128

[B95] Rozema J. Broekman R. A. Blokker P. Meijkamp B. B. de Bakker N. (2001). UV-B absorbance and UV-B absorbing compounds (para-coumaric acid) in pollen and sporopollenin: the perspective to track historic UV-B levels. *J. Photochem. Photobiol. B Biol.* 62 108–117. 10.1016/S1011-1344(01)00155-5 11693361

[B96] Ruan Y. L. Jin Y. Yang Y. J. Li G. J. Boyer J. S. (2010). Sugar input, metabolism, and signaling mediated by invertase: roles in development, yield potential, and response to drought and heat. *Mol. Plant* 3 942–955. 10.1093/mp/ssq044 20729475

[B97] Saika H. Okamoto M. Miyoshi K. Kushiro T. Shinoda S. Jikumaru Y. (2007). Ethylene promotes submergence-induced expression of OsABA8ox1, a gene that encodes ABA 8’-hydroxylase in rice. *Plant Cell Physiol.* 48 287–298. 10.1093/pcp/pcm003 17205969

[B98] Saini H. S. Sedgley M. Aspinall D. (1984). Development anatomy in wheat of male sterility induced by heat stress, water deficit or abscisic acid. *Funct. Plant Biol.* 11 243–253.

[B99] Saito H. Oikawa T. Hamamoto S. Ishimaru Y. Kanamori-Sato M. Sasaki-Sekimoto Y. (2015). The jasmonate-responsive GTR1 transporter is required for gibberellin-mediated stamen development in *Arabidopsis*. *Nat. Commun.* 6:6095 10.1038/ncomms7095 PMC4347201 25648767

[B100] Sakata T. Oda S. Tsunaga Y. Shomura H. Kawagishi-Kobayashi M. Aya K. (2014). Reduction of gibberellin by low temperature disrupts pollen development in rice. *Plant Physiol.* 164 2011–2019. 10.1104/pp.113.234401 24569847 PMC3982758

[B101] Schwartz S. H. Tan B. C. Gage D. A. Zeevaart J. A. McCarty D. R. (1997). Specific oxidative cleavage of carotenoids by VP14 of maize. *Science* 276 1872–1874. 10.1126/science.276.5320.1872 9188535

[B102] Seo M. Koshiba T. (2002). Complex regulation of ABA biosynthesis in plants. *Trends Plant Sci.* 7 41–48. 10.1016/S1360-1385(01)02187-2 11804826

[B103] Sharma K. D. (2014). Pollen development under cold stress: a molecular perspective. *Austin J. Genet. Genomic Res.* 1:4.

[B104] Sharma K. D. Nayyar H. (2014). Cold stress alters transcription in meiotic anthers of cold tolerant chickpea (*Cicer arietinum* L.). *BMC Res. Notes* 7:717 10.1186/1756-0500-7-717 PMC4201710 25306382

[B105] Sharma M. Laxmi A. (2015). Jasmonates: emerging players in controlling temperature stress tolerance. *Front. Plant Sci.* 6:1129 10.3389/fpls.2015.01129 PMC4701901 26779205

[B106] Shimono H. Abe A. Aoki N. Koumoto T. Sato M. Yokoi S. (2016). Combining mapping of physiological quantitative trait loci and transcriptome for cold tolerance for counteracting male sterility induced by low temperatures during reproductive stage in rice. *Physiol. Plant.* 10.1111/ppl.12410 26607766

[B107] Song S. Huang H. Gao H. Wang J. Wu D. Liu X. (2014). Interaction between MYC2 and ETHYLENE INSENSITIVE3 modulates antagonism between jasmonate and ethylene signaling in *Arabidopsis*. *Plant Cell* 26 263–279. 10.1105/tpc.113.120394 24399301 PMC3963574

[B108] Song S. Qi T. Huang H. Xie D. (2013). Regulation of stamen development by coordinated actions of jasmonate, auxin, and gibberellin in *Arabidopsis.* *Mol. Plant* 6 1065–1073. 10.1093/mp/sst054 23543439

[B109] Strauss G. Hauser H. (1986). Stabilization of lipid bilayer vesicles by sucrose during freezing. *Proc. Natl. Acad. Sci. U.S.A.* 83 2422–2426. 10.1073/pnas.83.8.2422 16593683 PMC323309

[B110] Tabata R. Ikezaki M. Fujibe T. Aida M. Tian C. E. Ueno Y. (2010). Arabidopsis auxin response factor6 and 8 regulate jasmonic acid biosynthesis and floral organ development via repression of class 1 KNOX genes. *Plant Cell Physiol.* 51 164–175. 10.1093/pcp/pcp176 20007966

[B111] Taji T. Ohsumi C. Iuchi S. Seki M. Kasuga M. Kobayashi M. (2002). Important roles of drought-and cold-inducible genes for galactinol synthase in stress tolerance in *Arabidopsis thaliana*. *Plant J.* 29 417–426. 10.1046/j.0960-7412.2001.01227.x 11846875

[B112] Takada K. Ishimaru K. Kamada H. Ezura H. (2006). Anther-specific expression of mutated melon ethylene receptor gene Cm-ERS1/H70A affected tapetum degeneration and pollen grain production in transgenic tobacco plants. *Plant Cell Rep.* 25 936–941. 10.1007/s00299-006-0147-0 16552596

[B113] Tang R. S. Zheng J. C. Jin Z. Q. Zhang D. D. Huang Y. H. Chen L. G. (2008). Possible correlation between high temperature-induced floret sterility and endogenous levels of IAA, GAs and ABA in rice (*Oryza sativa* L.). *Plant Growth Regulat.* 54 37–43. 10.1007/s10725-007-9225-8

[B114] Tarkowski Ł. P. Van den Ende W. (2015). Cold tolerance triggered by soluble sugars: a multifaceted countermeasure. *Front. Plant Sci.* 6:203 10.3389/fpls.2015.00203 PMC4396355 25926837

[B115] Teng K. Li J. Liu L. Han Y. Du Y. Zhang J. (2014). Exogenous ABA induces drought tolerance in upland rice: the role of chloroplast and ABA biosynthesis-related gene expression on photosystem II during PEG stress. *Acta Physiol. Plant.* 36 2219–2227. 10.1007/s11738-014-1599-4

[B116] Thakur P. Kumar S. Malik J. A. Berger J. D. Nayyar H. (2010). Cold stress effects on reproductive development in grain crops: an overview. *Environ. Exp. Bot.* 67 429–443. 10.1016/j.envexpbot.2009.09.004

[B117] Ueguchi-Tanaka M. Nakajima M. Katoh E. Ohmiya H. Asano K. Saji S. (2007). Molecular interactions of a soluble gibberellin receptor, GID1, with a rice DELLA protein, SLR1, and gibberellin. *Plant Cell* 19 2140–2155. 10.1105/tpc.106.043729 17644730 PMC1955699

[B118] Van den Ende W. El-Esawe S. K. (2014). Sucrose signaling pathways leading to fructan and anthocyanin accumulation: a dual function in abiotic and biotic stress responses? *Environ. Exp. Bot.* 108 4–13. 10.1016/j.envexpbot.2013.09.017

[B119] Vereyken I. J. Chupin V. Demel R. A. Smeekens S. C. De Kruijff B. (2001). Fructans insert between the headgroups of phospholipids. *Biochim. Biophys. Acta* 1510 307–320. 10.1016/S0005-2736(00)00363-1 11342168

[B120] Wang M. Hoekstra S. Van Bergen B. S. Lamers G. E. Oppedijk B. J. van der Heijden M. W. (1999). Apoptosis in developing anthers and the role of ABA in this process during androgenesis in *Hordeum vulgare* L. *Plant Mol. Biol.* 39 489–501. 10.1023/A:1006198431596 10092177

[B121] Wei M. Song M. Fan S. Yu S. (2013). Transcriptomic analysis of differentially expressed genes during anther development in genetic male sterile and wild type cotton by digital gene-expression profiling. *BMC Genomics* 14:1 10.1186/1471-2164-14-97 PMC3599889 23402279

[B122] Wilson Z. A. Zhang D. (2009). From *Arabidopsis* to rice. Pathways in pollen development. *J. Exp. Bot.* 60 1479–1492. 10.1093/jxb/erp095 19321648

[B123] Xu Z. Y. Lee K. H. Dong T. Jeong J. C. Jin J. B. Kanno Y. (2012). A vacuolar β-glucosidase homolog that possesses glucose-conjugated abscisic acid hydrolyzing activity plays an important role in osmotic stress responses in *Arabidopsis*. *Plant Cell* 24 2184–2199. 10.1105/tpc.112.095935 22582100 PMC3442595

[B124] Yang W. C. Ye D. Xu J. Sundaresan V. (1999). The SPOROCYTELESS gene of Arabidopsis is required for initiation of sporogenesis and encodes a novel nuclear protein. *Genes Dev.* 13 2108–2117. 10.1101/gad.13.16.2108 10465788 PMC316961

[B125] Yoshida T. Mogami J. Yamaguchi-Shinozaki K. (2014). ABA-dependent and ABA-independent signaling in response to osmotic stress in plants. *Curr. Opin. Plant Biol.* 21 133–139. 10.1016/j.pbi.2014.07.009 25104049

[B126] Yu C. Xu X. Ge J. Guo Y. Dong J. Dong Z. (2016). Premature breakdown of tapetum associated with reverse thermo-sensitive genic male-sterile line Huiyou50S in rapeseed (*Brassica napus*). *Acta Physiol. Plant.* 38 1–10. 10.1007/s11738-015-2039-9

[B127] Zanor M. I. Osorio S. Nunes-Nesi A. Carrari F. Lohse M. Usadel B. (2009). RNA interference of LIN5 in Solanum lycopersicum confirms its role in controlling Brix content, uncovers the influence of sugars on the levels of fruit hormones and demonstrates the importance of sucrose cleavage for normal fruit development and fertility. *Plant Physiol.* 150 1204–1218. 10.1104/pp.109.136598 19439574 PMC2705052

[B128] Zentella R. Hu J. Hsieh W. P. Matsumoto P. A. Dawdy A. Barnhill B. (2016). O-GlcNAcylation of master growth repressor DELLA by SECRET AGENT modulates multiple signaling pathways in *Arabidopsis*. *Genes Dev.* 30 164–176. 10.1101/gad.270587.115 26773002 PMC4719307

[B129] Zentella R. Yamauchi D. Ho T. H. (2002). Molecular dissection of the gibberellin/ abscisic acid signaling pathways by transiently expressed RNA interference in barley aleurone cells. *Plant Cell* 14 2289–2301. 10.1105/tpc.003376 12215521 PMC150771

[B130] Zhang D. Yang L. (2014). Specification of tapetum and microsporocyte cells within the anther. *Curr. Opin. Plant Biol.* 17 49–55. 10.1016/j.pbi.2013.11.001 24507494

[B131] Zhang H. Liang W. Yang X. Luo X. Jiang N. Ma H. (2010). Carbon starved anther encodes a MYB domain protein that regulates sugar partitioning required for rice pollen development. *Plant Cell* 22 672–689. 10.1105/tpc.109.073668 20305120 PMC2861464

[B132] Zhang X. Y. Wang X. L. Wang X. F. Xia G. H. Pan Q. H. Fan R. C. (2006). A shift of phloem unloading from symplasmic to apoplasmic pathway is involved in developmental onset of ripening in grapeberry. *Plant Physiol.* 142 220–232. 10.1104/pp.106.081430 16861573 PMC1557625

[B133] Zhang Y. Chen J. Liu J. Xia M. Wang W. Shen F. (2015). Transcriptome analysis of early anther development of cotton revealed male sterility genes for major metabolic pathways. *J. Plant Growth Regulat.* 34 223–232. 10.1007/s00344-014-9458-5

[B134] Zhang Y. Zhen L. Tan X. Li L. Wang X. (2014). The involvement of hexokinase in the coordinated regulation of glucose and gibberellin on cell wall invertase and sucrose synthesis in grape berry. *Mol. Biol. Rep.* 41 7899–7910. 10.1007/s11033-014-3683-7 25163631

[B135] Zhang Z. Huang R. (2010). Enhanced tolerance to freezing in tobacco and tomato overexpressing transcription factor TERF2/LeERF2 is modulated by ethylene biosynthesis. *Plant Mol. Biol.* 73 241–249. 10.1007/s11103-010-9609-4 20135196

[B136] Zhang Z. Wang Y. Chang L. Zhang T. An J. Liu Y. (2016). MsZEP, a novel zeaxanthin epoxidase gene from alfalfa (*Medicago sativa*), confers drought and salt tolerance in transgenic tobacco. *Plant Cell Rep.* 35 439–453. 10.1007/s00299-015-1895-5 26573680

[B137] Zhao M. Liu W. Xia X. Wang T. Zhang W. H. (2014). Cold acclimation-induced freezing tolerance of *Medicago truncatula* seedlings is negatively regulated by ethylene. *Physiol. Plant.* 152 115–129. 10.1111/ppl.12161 24494928

[B138] Zhu J. Zhang K. X. Wang W. S. Gong W. Liu W. C. Chen H. G. (2015). Low temperature inhibits root growth by reducing auxin accumulation via ARR1/12. *Plant Cell Physiol.* 56 727–736. 10.1093/pcp/pcu217 25552473

[B139] Zhu Z. An F. Feng Y. Li P. Xue L. Mu A. (2011). Derepression of ethylene-stabilized transcription factors (EIN3/EIL1) mediates jasmonate and ethylene signaling synergy in *Arabidopsis*. *Proc. Natl. Acad. Sci. U.S.A.* 108 12539–12544. 10.1073/pnas.1103959108 21737749 PMC3145709

